# 6-month SARS-CoV-2 antibody persistency in a Tyrolian COVID-19 cohort

**DOI:** 10.1007/s00508-020-01795-7

**Published:** 2020-12-09

**Authors:** Florian Deisenhammer, Wegene Borena, Angelika Bauer, Janine Kimpel, Dagmar Rudzki, Kathrin Schanda, Jonas Egeter, Katharina Hüfner, Barbara Sperner-Unterweger, Markus Reindl

**Affiliations:** 1 Department of Neurology, Neuroimmunology Laboratory, Medical University of Innsbruck, Innrain 66, 2nd floor, 6020 Innsbruck, Austria; 2 Institute of Virology, Department of Hygiene, Microbiology and Public Health, Medical University of Innsbruck, Innsbruck, Austria; 3 Department of Neurology, Medical University of Innsbruck, Innsbruck, Austria; 4 Division of Psychiatry II, Department of Psychiatry, Psychotherapy and Psychosomatics, Medical University of Innsbruck, Innsbruck, Austria

**Keywords:** Immunity, Prospective, ELISA, Virus, Neutralizing

## Abstract

**Background:**

As coronavirus disease 2019 caused by severe acute respiratory syndrome coronavirus 2 evolved only recently, the persistency of the anti-viral antibody response remains to be determined.

**Methods:**

We prospectively followed 29 coronavirus disease 2019 cases, mean age 44 ± 13.2 years. Except for one participant with a pre-existing diagnosis of rheumatoid arthritis, all other participants were previously healthy. We determined anti-viral binding antibodies at 2–10 weeks, 3 months, and 6 months after disease onset as well as neutralizing antibodies at 6 months. Two binding antibody assays were used, targeting the S1 subunit of the spike protein, and the receptor binding domain.

**Results:**

All participants fully recovered spontaneously except for one who had persisting hyposmia. Antibodies to the receptor binding domain persisted for 6 months in all cases with a slight increase of titers, whereas antibodies to S1 dropped below the cut-off point in 2 participants and showed a minimal decrease on average, mainly at month 3 of follow-up in males; however, neutralizing antibodies were detected in all samples at 6 months of follow-up.

**Conclusion:**

There is a stable and persisting antibody response against acute respiratory syndrome coronavirus 2 at 6 months after infection. Neutralizing antibodies confirm virus specificity. As the number of coronavirus disease 2019 convalescent cases is increasing sharply, antibody testing should be implemented to identify immunized individuals. This information can be helpful in various settings of professional and private life.

## Introduction

Coronavirus disease 2019 (COVID-19) caused by severe acute respiratory syndrome coronavirus 2 (SARS-CoV-2) emerged in Austria in early 2020 [1] receiving utmost attention in every aspect of private and public life. By 16 November 2020 more than 200,000 people were infected with SARS-CoV‑2 in Austria with more than 1700 deaths (https://covid19-dashboard.ages.at). In order to reduce spreading of the disease political decisions had to be made that severely affected the quality of life not only of Austrians but of all people worldwide and also has a substantial socioeconomic impact [2].

The SARS-CoV‑2 infection induces an immune response with activation of the innate and adaptive immune system leading to viral clearance and spontaneous recovery in the majority of nonfatal cases [3]. Virus-specific T and B cells evolve and consequently virus-specific antibodies are produced by plasma cells usually some days up to a few weeks after infection depending on the immunoglobulin (Ig) subclass [3]. Depending on the sample size, population, comorbidities, treatment, and type of antibody assay the vast majority of COVID-19 patients develop an antibody response [4–14].

The durability of the antibody response remains to be determined although short-term follow-up studies revealed stable antibody titers post-COVID-19 infection in symptomatic as well as asymptomatic cases [4, 6–10, 12–16].

This article presents 6 months follow-up SARS-CoV‑2 antibody results in a prospective Tyrolian cohort.

## Methods

### Study population

We identified a COVID-19 cluster at a grammar school in March 2020. Teachers who reported typical symptoms and/or had a positive SARS-CoV‑2 PCR test result as well as their partners and other household members were included. Furthermore, the layout of the school’s staffroom was assessed and colleagues who fulfilled contact criteria (i.e. sharing a desk with a COVID-19 case and regular presence in school during the week before shut-down on 15 March 2020) were also invited for antibody testing. Students attending classes of affected teachers were directly asked if COVID-19 has been diagnosed. Also, we inquired at the school’s directorate and Tyrolean health authorities if any affected students were reported. The school hosts roughly 700 students from 9th to 13th form, i.e. the typical age ranges from 15 to 19 years. The authors’ work environment (University Hospital Innsbruck) was screened including relatives of hospital staff members were included.

In total 169 persons were screened for SARS-CoV‑2 antibodies.

### Case definitions

A thorough history was taken from all participants and according to the European Centre for Disease Prevention and Control (www.ecdc.europa.eu/en/covid-19/surveillance/case-definition) COVID-19 cases were clinically defined as any person with at least one of the following symptoms: cough, fever, shortness of breath, sudden onset of anosmia, ageusia or dysgeusia.

The following case classification were applied:

- Possible case: any person meeting the clinical criteria
- Probable case: any person meeting the clinical criteria with an epidemiological link
- Confirmed case: any person meeting the laboratory criteria.

### Samples

For antibody assay verification we included samples as negative controls that were collected during the year 2020 from a previous unrelated study (historic controls) as well as current samples from potentially COVID-19 exposed persons (mostly hospital staff) who were asymptomatic. Also, 82 subjects who participated in a survey initiated by the Department of Psychiatry, Psychotherapy and Psychosomatics from whom blood was collected at a single time point after PCR testing were analyzed.

Negative controls consisted of 187 samples.

Positive controls (46 samples including patients who were not part of the prospective cohort) were taken from confirmed or probable COVID-19 cases with a minimum interval of 2 weeks after symptom onset.

### Prospective cohort

In COVID-19 cases as well as asymptomatic subjects who fulfilled the contact criteria and had a positive SARS-CoV‑2 antibody test in April 2020 at the latest, blood samples were serially collected at 3 time points after symptom onset. T1 between 2 weeks up to 2 months, T2 between 3 and 4 months, and T3 at 6 months. Binding SARS-CoV‑2 antibodies were determined at all time points and neutralizing antibodies were tested in all samples at T3.

### Assays

#### Detection of SARS-CoV-2 RNA by real-time PCR

Real-time (RT) PCR for the detection of SARS-CoV‑2 was performed using the RealStar® SARS-CoV‑2 RT-PCR kit 1.0 (Altona Diagnostics GmbH, Hamburg, Germany). Cycle threshold (Ct) values below 40 were rated as positive. The validity of the test was assured using negative, positive, and internal controls.

#### Anti-SARS-CoV-2 binding antibodies (ELISA)

We used two commercially available SARS-CoV‑2 antibody ELISA assays and performed tests according to the manufacturers’ instructions. One assay uses the SARS-CoV‑2 receptor binding domain (RBD) of the S1 subunit of the spike protein as target in a two-step incubation antigen sandwich enzyme immunoassay kit. Bound antibodies (total Ig) were detected using peroxidase labelled RBD (RBD pan-Ig; Wantai Biological, Bejing, China). This assay was previously evaluated by the Institute of Virology and AGES (https://www.ages.at/en/wissen-aktuell/publikationen/evaluierung-des-wantai-sars-cov-2-ab-rapid-tests/) with excellent sensitivity and specificity. The other assay uses the full S1 subunit of the spike protein as antigen and an anti-human IgG detector antibody (Euroimmun, Lübeck, Germany). Assay read-outs are optical densities (OD) and results are reported as index values, which are obtained by the ratio between the test sample OD and a reference sample OD provided with the test kit with a slight modification in the latter assay because the interassay precision of the provided reagents was unsatisfactory. Therefore, we calculated a cut-off OD from negative controls as the sample ODs had a much better performance in interassay precision (*see* results). Both assays are fully validated and CE-certified.

#### Anti-SARS-CoV-2 neutralizing antibody assay

The assay is based on a replication defective vesicular stomatitis virus (VSV) vector, which carries the glycoprotein of SARS-CoV‑2 in its envelope (VSV-S) as previously described [17].

This assay was validated by performing a neutralization test with the replication competent SARS-CoV‑2 isolated during the study under Biosafety level 3 (BSL3) conditions. Vectors were preincubated with patient samples and subsequently used for infection of Vero cells. Infectivity or neutralization was determined using a marker protein expressed by the VSV‑S vector (secreted alkaline phosphatase, SEAP) through ELISA-based colorimetric detection. Twofold serum dilutions of a 1:4 dilution were analyzed. The antibody dilution, which resulted in >50% reduction in the SEAP signal as compared to the negative control was defined as neutralizing titer.

### Statistics

Analyses were done using GraphPad Prism software version 8.41 (GraphPad Software, La Jolla, CA, USA).

Means, standard deviations, and ranges were calculated for normally distributed data. Nonparametric tests were used for nonnormally distributed data. Cohen’s kappa was determined for assay agreement, and Spearman coefficients for correlation analyses. For repeated measures, we applied Kruskal-Wallis test as there were 4 samples missing at T1 with adjusted *p*-values using Dunn’s multiple comparison test.

The tolerated type 1 error was set at 5%.

### Ethics

The study was approved by the local ethical committee and all participants gave written informed consent.

## Results

### Epidemiology

#### Infection rates in grammar school

The school’s index case (male, 43 years) attended a sports event in a crowded gym (approximately 300 participants) on 7 March 2020 and developed typical COVID-19 disease on 11 March 2020. There was no better explanation for the infection by any other sources. Until 18 March 2020 further 7 out of 80 teachers acquired symptomatic COVID-19, confirmed by SARS-CoV‑2 antibodies during this study, all of whom recovered spontaneously without sequalae resulting in an infection rate of 10% (95% confidence interval, CI 4–19%). Only two teachers were PCR tested during the symptomatic phase. The remainder were either declined by authorities or did not see a doctor because of mildness of symptoms. The average age was 51 years (range 38–46 years) comprising 5 males and 3 females. The typical desk space in the staffroom is 65–80 cm wide and the assigned workplaces of all affected teachers were close to each other (i.e. less than 2 m).

There are 8 teachers including the index case who meet regularly for coffee breaks in a separate room on a table which is 120×130 cm in size. Of those, 3 were affected by COVID-19 which comes down to a 38% (95% CI 9–76%) infection rate of this particular group.

Of the remaining 29 teachers (17 female 12 male, mean age 47 years, range 29–64 years) sharing desks with the COVID-19 cases, including the director who had regular contact to all staff members, all tested SARS-CoV‑2 antibody negative. Of those, 13 reported unspecific symptoms that occurred mostly during January and February 2020 and were therefore not considered possible COVID-19 cases.

To the best of our knowledge none of the approximately 700 students were affected until reopening of school in June 2020.

### Households and partnerships

We identified 16 couples, 1 single living with both parents, and 12 children living in the households of 5 of the couples. One partner in each of the couples contracted COVID-19 elsewhere (7 of those in school). Of the other partners 12 contracted the disease in the partnership and 4 were asymptomatic but 1 was SARS-CoV‑2 antibody positive. Of the children five reported typical symptoms but tested negative in the antibody assay. The two parents were asymptomatic. Including the antibody positive partner, the infection rate in couples was 82% (95% CI 54–96%).

In this cohort 29 individuals (Table 1) developed SARS-CoV‑2 antibodies and were followed for 6 months. All cases fully recovered from COVID-19 symptoms except for one person in whom there is incomplete recovery of hyposmia to date. One participant has been diagnosed with rheumatoid arthritis (RA) and treated with azathioprine. All others were healthy at the time of COVID-19 onset. The RA patient experienced a relapse during COVID-19 disease which was treated with corticosteroids and resolved thereafter.

**Table 1:** Prospective cohort COVID-19 cases and contact persons by antibody status

	Antibody positive (*n* = 29)	Antibody negative (*n* = 46)
*Female/male*	14/15	28/18
*Age (years, mean* *±* *SD)*	44 ± 13.2	39 ± 16.8
*Cases by classification*		
Confirmed (*n*)	13	0
Probable (*n*)	15	8
Asymptomatic (*n*)	1	38

One of the SARS-CoV‑2 antibody positive participants was re-exposed as the partner contracted PCR-confirmed COVID-19 recently. The participant had no symptoms at the second exposure and tested negative on SARS-CoV‑2 PCR.

### SARS-CoV-2 binding antibody assay performance

Diagnostic sensitivity was 91.5% for the S1 IgG assay and 95.5% for the RBD pan-Ig assay. Diagnostic specificity was 97.7% for the S1 IgG assay and 97.4% for the RBD pan-Ig assay.

The average interassay precision was 34% for the reagents provided by Euroimmun (positive and negative controls, calibrator) and 5% for the sample ODs. The interassay precision for the positive control of the RBD pan-Ig assay (Wantai) was 15%, for the sample ODs 9%, and 12% for the calculated OD index values. Because of the low performance of the Euroimmun reagents we determined an upper reference limit of normal by adding 3 times the standard deviation to the mean OD of 122 negative controls. In analogy to the RBD pan-Ig assay, we then calculated the S1 IgG index values by dividing sample ODs by the reference OD.

Of 167 samples from the prospective and retrospective (verification) cohorts tested in both ELISAs, 163 (97.6%) had concordant results as shown in Table 2 with a kappa value for agreement of 0.950 (95% CI 0.901–0.998).

**Table 2:** SARS-CoV‑2 binding antibody assay agreement

		S1 IgG		
		Pos	Neg	Total
*RBD pan-Ig*	*Pos*	99	3	102
	*Neg*	1	64	65
	*Total*	100	67	167

Cohen’s kappa = 0.950 (95% confidence interval [CI] 0.901–0.998)

### SARS-CoV-2 antibody persistency

In the prospective cohort 29 individuals were followed of whom 24 had blood collected at T1 (mean of 7 ± 2 weeks) and all 29 participants at T2 (mean of 14 ± 2 weeks), and T3 (mean of 27 ± 1 weeks).

**Fig. 1:**
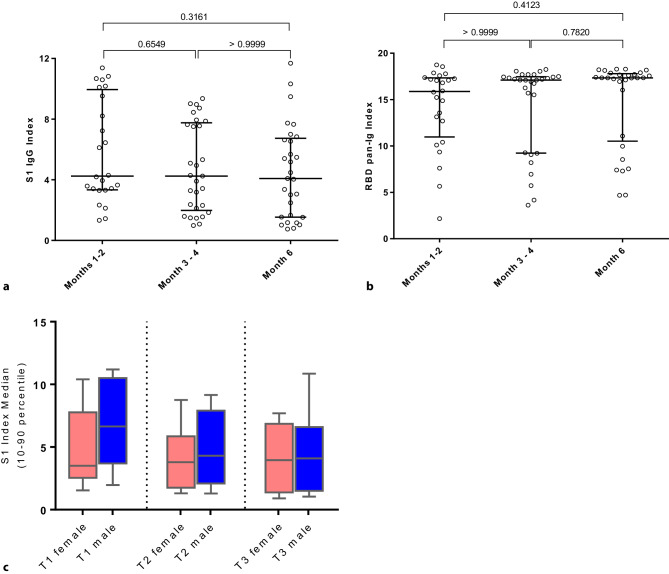
Anti-SARS-CoV‑2 antibody index values over time. **a** Antibody index values for the S1 IgG assay at 3 consecutive time points. Lines indicate median values and error bars show interquartile ranges. Corrected *p*-values are shown above the brackets. Spearman correlation coefficients for T1 vs. T2 was 0.89, and for T1 vs. T3 it was 0.70 (*p*-value <0.001 for both). Of note, at T1 (months 1–2) data of 4 individuals are missing. In each column one patient occurs only once. **b** Antibody index values for the RBD pan-Ig assay at three consecutive time points. Lines indicate median values and error bars show interquartile ranges. Corrected *p*-values are shown above the brackets. Spearman correlation coefficients for T1 vs. T2 was 0.65, and for T1 vs. T3 it was 0.49 (*p*-value <0.02 for both). Of note, at T1 (months 1–2) data of 4 individuals are missing. In each column one patient occurs only once. **c** S1 IgG indices by sex. Levels remained stable in females with median (95% CI) values at T1, T2 and T3 of 3.5 (2.3–8.2), 3.8 (1.6–7.5) and 3.95 (1.0–7.0), respectively, whereas a decline was observed in males with values at T1, T2 and T3 of 6.7 (3.6–10.6), 4.3 (2.1–7.9), and 4.1 (1.5–6.6), respectively. None of the differences were significant

**Fig. 2:**
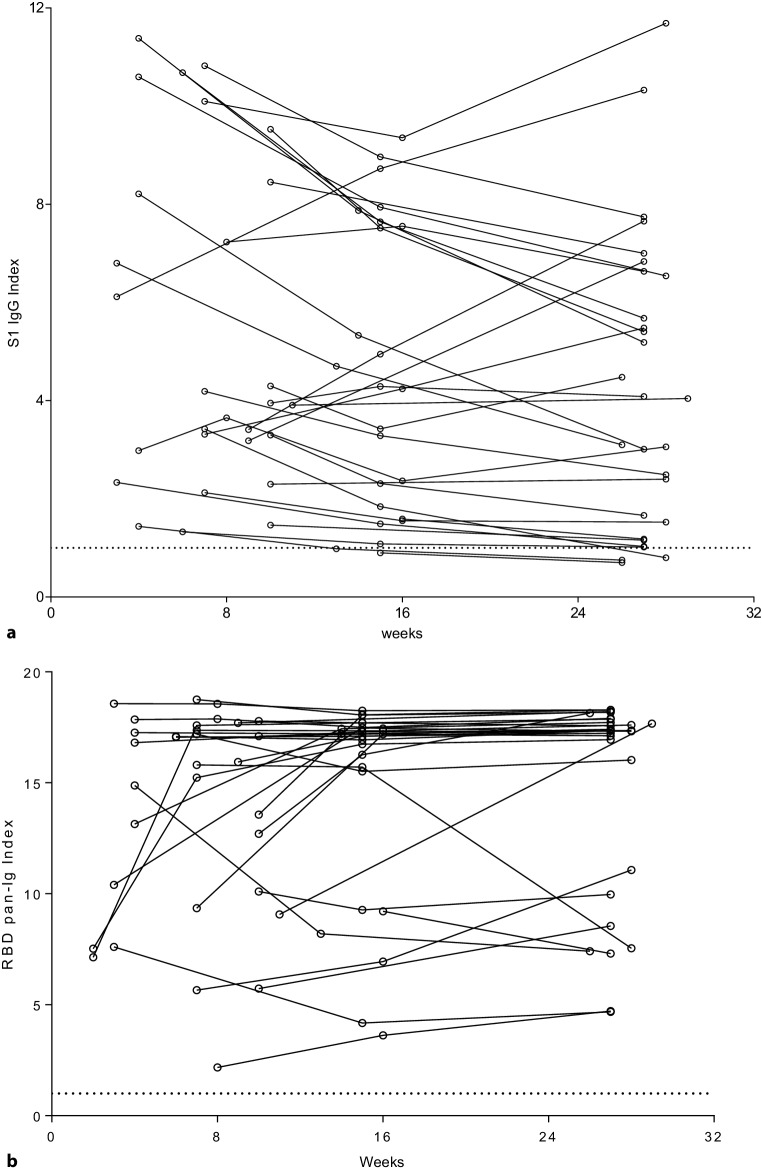
Individual time course of S1 IgG and RBD pan-Ig antibody indices. **a** Individual longitudinal S1 IgG index values. The dashed horizontal line indicates the cut-off point between negative and positive results. **b** Individual longitudinal RBD pan-Ig index values. The dashed horizontal line indicates the cut-off point between negative and positive results

At T2 one sample was negative in the S1 IgG assay but all samples were positive in the pan Ig assay. At T3 two samples (one of those already negative at T2) were negative in the S1 IgG assay and again all samples remained positive in the pan Ig assay (Figs. 1 and 2). Index values by the three time points are shown in Fig. 1a, b. There was no significant difference between these three time points regarding both S1 IgG and RBD pan-Ig values. The decline in S1 IgG indices was driven by male participants in the first 3 months after disease onset. This effect did not occur in females (Fig. 1c).

Plots of individual index values of all longitudinal samples are shown in Fig. 2.

At 6 months follow-up all participants had neutralizing anti-SARS-CoV‑2 antibodies with a median titer of 1:64 ranging from 1:16 to 1:256. Spearman correlation coefficients were 0.68 between neutralizing titers and S1 IgG index, and 0.69 between neutralizing titers and RBD pan-Ig values (*p* < 0.0001 for both).

## Discussion

In the present study we found a persistent anti-SARS-CoV‑2 antibody response over 6 months including neutralizing activity in all participants. The epidemiology in the subcohort of high school teachers as well as in household members followed known patterns with physical vicinity and time of exposure being the driving factors of transfection rates [18].

We feel that these data are important and reassuring for long-term immunity after COVID-19 disease. There is little doubt that there is protective immunity in convalescent cases. The immune response follows standard patterns of anti-viral protective immunity with neutralizing antibodies in the majority of infected people [7, 12, 15, 16, 19]. The SARS-CoV‑2 specific T‑cells and B‑cells have been demonstrated in patients recovered from COVID-19 as well as SARS-CoV‑2 specific memory cells [20]. In a large outbreak of COVID-19 on a fishery vessel affecting 104 out of 122 crew members the individuals with neutralizing anti-SARS-CoV‑2 antibodies remained asymptomatic and repeatedly tested negative in SARS-CoV‑2 PCR except for one person with a very weak PCR signal [19]. One of our antibody positive study participants remained asymptomatic and PCR negative after re-exposure through the partner, a situation with a high likelihood of infection as shown in the partnership analyses with a transfection rate of 82%. In nonhuman primates antibody-associated protective immunity could be demonstrated by SARS-CoV‑2 infection and reinfection [21]. The animals had no COVID-19 symptoms after re-inoculation and a boost of their antibody response.

There are a few reports of possible SARS-CoV‑2 reinfection [22–24]; however, no case occurred in a similar setting to our study population, i.e. reinfection after a reasonably stable phase of convalescence and persisting antibody response. Most of the reported reinfections were most likely reactivations shortly after primary disease onset or had no antibody response after the first event, a notion supported by a recent study on a larger population concluding that reoccurrence of viral RNA after COVID-19 recovery reflects reactivation rather than true reinfection as viral clearance may take up to 3 months after primary infection [25].

The technical assay performance was good with sensitivities and specificities very similar to previous reports [26] and a slight advantage for the RBD pan-Ig assay, which seems to be more sensitive and stable over time [7, 8, 12]. The decline of the anti-S1 IgG response has been described earlier [4], it is however, unclear how to interpret this finding. Both subjects who had S1 IgG indices below cut-off point at 6 months still had neutralizing activity and a positive response against the RBD. It seems that the S1 protein harboring the receptor binding domain is the main target for virus neutralization [27], corroborated by the strong correlation between binding and neutralizing titers found by us and others [16]. We assume that epitopes are expressed differently on RBD versus full S1 protein. Also, the assay formats differ with direct coating of S1 onto the plate for the Euroimmun assay as opposed to a sandwich design provided by the Wantai test. The difference in antibody kinetics were also observed previously [4]. Apart from the present study, we are aware of only one other report following COVID-19 cases for 6 months showing persisting neutralizing anti-SARS-CoV‑2 antibodies in all post-COVID-19 cases after 6–7 months along with T‑cell memory [15].

Of course, one can only speculate about the future persistency of antibodies but the kinetics so far seems promising for a long-lasting response which depends on titers as indicated by the strong correlation between baseline and follow-up index values. Also, the curve of decline flattens. We found the difference of S1 IgG index values to be larger between T1 and T2, an effect that was restricted to males, whereas hardly any changes occurred later. Males are usually more severely affected by the disease and might therefore display a larger boost in the first weeks of their antibody response [28]. Several other studies found similar results pointing towards a long-lasting immune response [4, 15, 16].

The limitations that need to be mentioned include the relatively low number of study participants; however, this is one of the few prospective cohorts followed for 6 months. Although few studies found an early decline of antibody levels [29] the majority of reports are much in line with our findings. In this respect, one has to consider the many different assays that were used by various investigators and might account for discordant results. Furthermore, our results apply to a largely healthy population aged between 29 and 65 years with mild to moderate COVID-19 disease. Therefore, we cannot speak for children, an older population, immunocompromized, and people with relevant comorbidities who might behave very differently in terms of immunity and duration thereof.

In practical terms we suggest testing all convalescent cases for antibodies a few weeks after recovery and performing follow-up tests every 3–6 months depending on the titer. Any validated S1 or RBD binding assay should suffice as there is strong evidence that these assays are a surrogate for neutralization. Given the rapidly increasing number of people recovered from COVID-19, sooner rather than later the status of immunity needs to be acknowledged, which can be applied in many different contexts. Firstly, there is no need for vaccination in already immunized persons. Also, scheduling in healthcare deployment, particularly for nurses who are frequently in close contact with patients, could consider immunity and in fact any other professions where deployment plans are needed. In workplaces, where close contact between employees occur, such as distribution centers, vessels, or meat industry knowledge of immunity can be helpful [19, 30]. In fact, in these environments COVID-19 clusters occurred in the past resulting in many immunized people who can now stay together for work with very low risk of new clusters for the time being.

## References

[1] Kreidl P, Schmid D, Maritschnik S, Richter L, Borena W, Genger JW (2020). Emergence of coronavirus disease 2019 (COVID-19) in Austria. Wien Klin Wochenschr.

[2] Mofijur M, Fattah IMR, Alam MA, Islam ABMS, Ong HC, Rahman SMA (2021). Impact of COVID-19 on the social, economic, environmental and energy domains: Lessons learnt from a global pandemic. Sustain Prod Consum.

[3] Vabret N, Britton GJ, Gruber C, Hegde S, Kim J, Kuksin M (2020). Immunology of COVID-19: current State of the Science. Immunity.

[4] Gudbjartsson DF, Norddahl GL, Melsted P, Gunnarsdottir K, Holm H, Eythorsson E (2020). Humoral immune response to SARS-CoV-2 in iceland. N Engl J Med.

[5] Zhao J, Yuan Q, Wang H, Liu W, Liao X, Su Y (2020). Antibody responses to SARS-CoV-2 in patients with novel Coronavirus disease 2019. Clin Infect Dis.

[6] Long QX, Liu BZ, Deng HJ, Wu GC, Deng K, Chen YK (2020). Antibody responses to SARS-CoV-2 in patients with COVID-19. Nat Med.

[7] Premkumar L, Segovia-Chumbez B, Jadi R, Martinez DR, Raut R, Markmann A (2020). The receptor binding domain of the viral spike protein is an immunodominant and highly specific target of antibodies in SARS-CoV-2 patients. Sci Immunol.

[8] Amanat F, Stadlbauer D, Strohmeier S, Nguyen THO, Chromikova V, McMahon M (2020). A serological assay to detect SARS-CoV-2 seroconversion in humans. Nat Med.

[9] Liu L, To KK, Chan KH, Wong YC, Zhou R, Kwan KY (2020). High neutralizing antibody titer in intensive care unit patients with COVID-19. Emerg Microbes Infect.

[10] Chen Y, Zuiani A, Fischinger S, Mullur J, Atyeo C, Travers M (2020). Quick COVID-19 healers sustain anti-SARS-CoV-2 antibody production. Cell.

[11] Long QX, Tang XJ, Shi QL, Li Q, Deng HJ, Yuan J (2020). Clinical and immunological assessment of asymptomatic SARS-CoV-2 infections. Nat Med.

[12] Iyer AS, Jones FK, Nodoushani A, Kelly M, Becker M, Slater D (2020). Persistence and decay of human antibody responses to the receptor binding domain of SARS-CoV-2 spike protein in COVID-19 patients. Sci Immunol.

[13] Isho B, Abe KT, Zuo M, Jamal AJ, Rathod B, Wang JH (2020). Persistence of serum and saliva antibody responses to SARS-CoV-2 spike antigens in COVID-19 patients. Sci Immunol.

[14] Ma H, Zeng W, He H, Zhao D, Jiang D, Zhou P (2020). Serum IgA, IgM, and IgG responses in COVID-19. Cell Mol Immunol.

[15] Tan Y, Liu F, Xu X, Ling Y, Huang W, Zhu Z, et al. Durability of neutralizing antibodies and T‑cell response post SARS-CoV‑2 infection. Front Med. 2020:eabe5511. 10.1007/s11684-020-0822-5 10.1007/s11684-020-0822-5 PMC7533664 33017040

[16] Wajnberg A, Amanat F, Firpo A, Altman DR, Bailey MJ, Mansour M, et al. Robust neutralizing antibodies to SARS-CoV‑2 infection persist for months. Science. 2020:eabd7728. 10.1126/science.abd7728 10.1126/science.abd7728 PMC7810037 33115920

[17] Hoffmann M, Kleine-Weber H, Schroeder S, Kruger N, Herrler T, Erichsen S (2020). SARS-coV-2 cell entry depends on ACE2 and TMPRSS2 and is blocked by a clinically proven protease inhibitor. Cell.

[18] Ng OT, Marimuthu K, Koh V, Pang J, Linn KZ, Sun J (2020). SARS-CoV-2 seroprevalence and transmission risk factors among high-risk close contacts: a retrospective cohort study. Lancet Infect Dis.

[19] Addetia A, Crawford KHD, Dingens A, Zhu H, Roychoudhury P, Huang ML (2020). Neutralizing antibodies correlate with protection from SARS-CoV-2 in humans during a fishery vessel outbreak with high attack rate. J Clin Microbiol.

[20] Peng Y, Mentzer AJ, Liu G, Yao X, Yin Z, Dong D (2020). Broad and strong memory CD4(+) and CD8(+) T cells induced by SARS-CoV-2 in UK convalescent individuals following COVID-19. Nat Immunol.

[21] Deng W, Bao L, Liu J, Xiao C, Liu J, Xue J (2020). Primary exposure to SARS-CoV-2 protects against reinfection in rhesus macaques. Science.

[22] Gousseff M, Penot P, Gallay L, Batisse D, Benech N, Bouiller K (2020). Clinical recurrences of COVID-19 symptoms after recovery: viral relapse, reinfection or inflammatory rebound?. J Infect.

[23] Duggan NM, Ludy SM, Shannon BC, Reisner AT, Wilcox SR (2020). Is novel coronavirus 2019 reinfection possible? Interpreting dynamic SARS-CoV-2 test results through a case report. Am J Emerg Med.

[24] Tillett RL, Sevinsky JR, Hartley PD, Kerwin H, Crawford N, Gorzalski A (2020). Genomic evidence for reinfection with SARS-CoV-2: a case study. Lancet Infect Dis.

[25] Yang C, Jiang M, Wang X, Tang X, Fang S, Li H (2020). Viral RNA level, serum antibody responses, and transmission risk in recovered COVID-19 patients with recurrent positive SARS-CoV-2 RNA test results: a population-based observational cohort study. Emerg Microbes Infect.

[26] Bailey D, Konforte D, Barakauskas VE, Yip PM, Kulasingam V, El bou HM (2020). Canadian society of clinical chemists (CSCC) interim consensus guidance for testing and reporting of SARS-CoV-2 serology. Clin Biochem.

[27] Amanat F, Krammer F (2020). SARS-CoV-2 vaccines: status report. Immunity.

[28] Kowitdamrong E, Puthanakit T, Jantarabenjakul W, Prompetchara E, Suchartlikitwong P, Putcharoen O (2020). Antibody responses to SARS-CoV-2 in patients with differing severities of coronavirus disease 2019. PLoS ONE.

[29] Bruni M, Cecatiello V, az-Basabe A, Lattanzi G, Mileti E, Monzani S (2020). Persistence of anti-SARS-CoV-2 antibodies in non-hospitalized COVID-19 convalescent health care workers. J Clin Med.

[30] Gunther T, Czech-Sioli M, Indenbirken D, Robitaille A, Tenhaken P, Exner M (2020). SARS-CoV-2 outbreak investigation in a German meat processing plant. EMBO Mol Med.

